# Abscess of the Antrum of Highmore

**Published:** 1893-04

**Authors:** 


### SELECTIONS.

### Abscess of the Antrum of Highmore.

J. M. Hunt in the *Liverpool Medico-Chirurgical Journal*, presents some important points for consideration in an interesting paper on this disease, usually so difficult of early diagnosis.

In the author's experience the so-called classical symptoms, (1) distention of the antrum, (2) swelling of the cheek, (3) infra-orbital pain, (4) escape of pus on lying on the sound side, are, as a rule, conspicuous by their absence. The one constant and all-important symptom is the presence of a purulent nasal discharge coming from the concavity of the middle turbinate, and escaping either by the anterior or posterior naris. Pain is, as a rule, present, and is generally intermittent in nature and supra-orbital in position. As regards the intra-nasal condition, diffuse hypertrophy of the middle turbinate, polypoid degeneration or even true polypi in the middle meatus, hypertrophy of the mucous membrane in the neighborhood of the hiatus semilunaris, bare bone in the middle turbinate or at the ostium maxillare are to be found in most cases. The author regards it as justifiable to open the antrum in any patient with unilateral purulent discharge coming from the concavity of the middle turbinate in its anterior half, and constantly or at times ill smelling, if there be no obvious cause for the discharge inside the nose itself, such as a foreign body or specific ulceration. He does not regard the trans-illumination of the antral cavities by means of Voltolini's electric lamp placed in the mouth as of much practical importance, although in a doubtful case it may at times be useful. Exploratory puncture carried out through the middle or inferior meatus with a Pravaz syringe or a Lichtwitz trochar and canula is a more reliable method. For purposes of treatment the best plan is to open the antrum from the alveolar process either through the socket of a tooth which has been removed or from the alveolar border.

